# Ischemic Stroke despite Oral Anticoagulant Therapy in Patients with Atrial Fibrillation

**DOI:** 10.1002/ana.25700

**Published:** 2020-02-27

**Authors:** David J. Seiffge, Gian Marco De Marchis, Masatoshi Koga, Maurizio Paciaroni, Duncan Wilson, Manuel Cappellari, Kosmas Macha, MD, Georgios Tsivgoulis, Gareth Ambler, Shoji Arihiro, Leo H. Bonati, Bruno Bonetti, Bernd Kallmünzer, Keith W. Muir, Paolo Bovi, Henrik Gensicke, Manabu Inoue, Stefan Schwab, Shadi Yaghi, Martin M. Brown, Philippe Lyrer, Masahito Takagi, Monica Acciarrese, Hans Rolf Jager, Alexandros A. Polymeris, Kazunori Toyoda, Michele Venti, Christopher Traenka, Hiroshi Yamagami, Andrea Alberti, Sohei Yoshimura, Valeria Caso, Stefan T. Engelter, David J. Werring, the RAF, RAF‐DOAC, CROMIS‐2, SAMURAI, NOACISP, Erlangen, and Verona registry collaborators

**Affiliations:** ^1^ Stroke Research Center, Department of Brain Repair and Rehabilitation University College London Queen Square Institute of Neurology, and the National Hospital for Neurology and Neurosurgery Queen Square London United Kingdom; ^2^ Neurology and Stroke Center, Department of Clinical Research University Hospital of Basel, University of Basel Basel Switzerland; ^3^ Department of Neurology and Stroke Center University Hospital of Bern Bern Switzerland; ^4^ Department of Cerebrovascular Medicine National Cerebral and Cardiovascular Center Suita Japan; ^5^ Stroke Unit and Division of Cardiovascular Medicine University of Perugia Perugia Italy; ^6^ Stroke Unit, Department of Neuroscience University Hospital Integrated Trust of Verona Verona Italy; ^7^ Department of Neurology University of Erlangen‐Nürnberg Erlangen Germany; ^8^ Second Department of Neurology National and Kapodistrian University of Athens School of Medicine, Attikon University Hospital Athens Greece; ^9^ Department of Neurology University of Tennessee Health Science Center Memphis TN; ^10^ Department of Statistical Science University College London London United Kingdom; ^11^ Institute of Neuroscience and Psychology, University of Glasgow, Queen Elizabeth University Hospital Glasgow United Kingdom; ^12^ Neurorehabilitation Unit University Center for Medicine of Aging and Rehabilitation Basel, Felix Platter Hospital, University of Basel Basel Switzerland; ^13^ NYU Langone Health New York NY; ^14^ Neuroradiological Academic Unit, Department of Brain Repair and Rehabilitation University College London Queen Square Institute of Neurology London United Kingdom

## Abstract

**Objective:**

It is not known whether patients with atrial fibrillation (AF) with ischemic stroke despite oral anticoagulant therapy are at increased risk for further recurrent strokes or how ongoing secondary prevention should be managed.

**Methods:**

We conducted an individual patient data pooled analysis of 7 prospective cohort studies that recruited patients with AF and recent cerebral ischemia. We compared patients taking oral anticoagulants (vitamin K antagonists [VKA] or direct oral anticoagulants [DOAC]) prior to index event (OAC_prior_) with those without prior oral anticoagulation (OAC_naive_). We further compared those who changed the type (ie, from VKA or DOAC, vice versa, or DOAC to DOAC) of anticoagulation (OAC_changed_) with those who continued the same anticoagulation as secondary prevention (OAC_unchanged_). Time to recurrent acute ischemic stroke (AIS) was analyzed using multivariate competing risk Fine–Gray models to calculate hazard ratios (HRs) and 95% confidence intervals (CIs).

**Results:**

We included 5,413 patients (median age = 78 years [interquartile range (IQR) = 71–84 years]; 5,136 [96.7%] had ischemic stroke as the index event, median National Institutes of Health Stroke Scale on admission = 6 [IQR = 2–12]). The median CHA_2_DS_2_‐Vasc score (congestive heart failure, hypertension, age≥ 75 years, diabetes mellitus, stroke/transient ischemic attack, vascular disease, age 65–74 years, sex category) was 5 (IQR = 4–6) and was similar for OAC_prior_ (n = 1,195) and OAC_naive_ (n = 4,119, *p* = 0.103). During 6,128 patient‐years of follow‐up, 289 patients had AIS (4.7% per year, 95% CI = 4.2–5.3%). OAC_prior_ was associated with an increased risk of AIS (HR = 1.6, 95% CI = 1.2–2.3, *p* = 0.005). OAC_changed_ (n = 307) was not associated with decreased risk of AIS (HR = 1.2, 95% CI = 0.7–2.1, *p* = 0.415) compared with OAC_unchanged_ (n = 585).

**Interpretation:**

Patients with AF who have an ischemic stroke despite previous oral anticoagulation are at a higher risk for recurrent ischemic stroke despite a CHA_2_DS_2_‐Vasc score similar to those without prior oral anticoagulation. Better prevention strategies are needed for this high‐risk patient group. ANN NEUROL 2020;87:677–687

Oral anticoagulation substantially reduces the risk for ischemic stroke and systemic embolism in patients with atrial fibrillation (AF). Nevertheless, patients with AF may still have an ischemic stroke despite taking oral anticoagulants.^1^ This is often regarded as a treatment failure, whose mechanisms include incompliance, reduced pharmacological efficacy of the anticoagulant in individual patients, or other factors such as alternative stroke mechanisms (eg, small vessel occlusion).^2^ These patients might be at particularly high risk of further ischemic stroke events, but this has not been investigated. Moreover, the optimal prevention strategy to reduce further recurrence risk in such patients is unknown. Direct oral anticoagulants (DOAC) are a proven effective alternative to vitamin K antagonists (VKA) for oral anticoagulation in patients with AF.^3^ Among patients with an ischemic stroke despite anticoagulant therapy, it is unknown whether changing the type of anticoagulant (VKA to DOAC, DOAC to VKA, or switching to an alternative DOAC) reduces the risk of recurrence.

We aimed to answer the following questions in patients with AF and an index acute ischemic stroke or transient ischemic attack (TIA). First, are patients who have an ischemic stroke or TIA despite taking oral anticoagulants (VKA or DOAC) at increased risk of recurrent acute ischemic stroke (AIS) or other outcome events? Second, after the index event, is changing the type of anticoagulant (VKA or DOAC or type of DOAC) associated with reduced rates of AIS?

## Patients and Methods

### *Study Design and Study Population*

We used pooled individual patient data from an established international collaboration of investigator‐initiated prospective cohort studies.^4^ The following studies were included: the single‐center prospective cohort studies from Verona, Italy,^5^ Erlangen, Germany,^6^ and Basel, Switzerland (Novel Oral Anticoagulants in Stroke Patients [NOACISP])^7^; the multicenter cohort studies Early Recurrence and Cerebral Bleeding in Patients with Acute Ischemic Stroke and Atrial Fibrillation (RAF^8^ and RAF‐NOAC^9^; 29 centers in Europe, Asia, and North America); the Clinical Relevance of Microbleeds in Stroke study (CROMIS‐2; 79 centers in the United Kingdom and 1 in The Netherlands)^10^, ^11^; and the Stroke Acute Management with Urgent Risk‐Factor Assessment and Improvement Study on Anticoagulant Therapy in Nonvalvular Atrial Fibrillation (SAMURAI‐NVAF, 18 centers in Japan).^12^, ^13^ Details about the participating studies can be obtained from the published studies^4^, ^5^, ^6^, ^7^, ^8^, ^9^, ^10^, ^11^, ^12^, ^14^ and prior publications.^4^

### *Inclusion and Exclusion Criteria*

We included patients with: (1) recent ischemic stroke or TIA; (2) diagnosis of nonvalvular AF either known prior to the index event or detected after the event; (3) information on anticoagulation therapy prior to and after index event available; and (4) systematic follow‐up for at least 3 months or longer after the index event for the presence or absence of recurrent AIS, intracerebral hemorrhage, and death. We excluded patients with: (1) mechanical heart valves; (2) rheumatic or severe mitral valve stenosis; or (3) missing information on antithrombotic treatment before the index event.

### *Data Collection and Baseline Data*

Data were collected as described in prior publications^15^, ^16^: briefly, local investigators filled in standardized forms with predefined variables using individual patient data from their corresponding study database. Completed forms were collected at the coordinating center in Basel, Switzerland, where the pooled analysis was performed. The corresponding author (DJS) had full access to all the data in the study and takes responsibility for its integrity and the data analysis.

### *Baseline Data*

The following baseline variables were recorded and provided by the participating studies: age, sex, type of index event (AIS or TIA); antithrombotic treatment (no treatment, antiplatelet agents, VKA or DOAC including type of DOAC) before and after index event; international normalized ration (INR) at index event (if patient was on VKA therapy); time from index event to first dose of VKA or DOAC; stroke severity on admission as assessed by the National Institutes of Health Stroke Scale (NIHSS)^17^; and use of intravenous thrombolysis or endovascular reperfusion therapy for index stroke. DOAC therapy was defined as one of the following drugs and dosages: apixaban 2.5mg or 5mg twice daily; dabigatran 110mg or 150mg twice daily; edoxaban 30mg or 60mg once daily; or rivaroxaban 15mg or 20mg once daily (10mg or 20mg once daily in Japan, according to the results from a domestic trial^18^). VKA therapy was defined as treatment with phenprocoumon (NOACISP, Erlangen) or acenocoumarol/warfarin (SAMURAI‐NVAF, RAF/RAF‐DOAC, CROMIS‐2, and Verona). The choice of treatment was decided by the treating physician.

The following risk factors were collected: history of ischemic stroke; history of intracranial hemorrhage; diabetes mellitus; hypertension; hypercholesterolemia; diagnosis of AF (known before stroke vs diagnosed after stroke)^19^, ^20^, ^21^; renal function (creatinine clearance [CrCl] in ml/min using the Chronic Kidney Disease Epidemiology Collaboration [CKD‐EPI] equation,^22^ which was further classified into normal renal function defined as CrCl > 50ml/min, modest renal failure defined as CrCl = 30–50ml/min, and severe kidney failure defined as CrCl < 30ml/min); current smoking; the CHA_2_DS_2_‐Vasc score (congestive heart failure, hypertension, age ≥ 75 years, diabetes mellitus, stroke/transient ischemic attack, vascular disease, age 65–74 years, sex category)^23^; and the HAS‐BLED score (hypertension, abnormal renal/liver function, stroke, bleeding history or predisposition, labile international normalized ratio, elderly, drugs/alcohol)^24^ designed to predict future AIS and major bleeding complications, respectively.

### *Follow‐Up*

All patients were followed up for at least 3 months after index event; in some studies, follow‐up data for up to 5.4 years after index event were available. During follow‐up, occurrence of the following outcome events was assessed: (1) recurrent AIS defined as new neurological symptoms and evidence for ischemic stroke on computed tomography (CT) or magnetic resonance imaging (MRI); (2) intracerebral hemorrhage (ICH) defined as new neurological symptoms associated with the detection of intracerebral hemorrhage on CT or MRI as defined as part of the International Society on Thrombosis and Haemostasis criteria^25^; and (3) all‐cause mortality (including fatal stroke). For patients on VKA, INR at outcome event (AIS or ICH) was collected if available. If available, information on time in therapeutic range for patients on VKA during follow‐up was collected, dichotomized as poor (<60%) and good (>60%). Among patients taking DOAC, self‐reported adherence was dichotomized as fully adherent (no missing dosage since last study visit) or nonadherent (at least 1 missing dosage since last study visit) based on information provided by the patient.^26^

### *Outcome*

The primary outcome of this analysis was recurrent AIS. Secondary endpoints were symptomatic ICH and mortality.

### *Ethics*

The NOACISP LONG‐TERM registry and the current analysis of pooled individual patient data were approved by the ethics committee in Basel, Switzerland (EKNZ 2014‐027). Patients provided written consent for participation in NOACISP LONG‐TERM. The requirement for additional local ethical approval differed among participating centers and was acquired by the local principal investigator as well as written informed consent by the patient if necessary. The SAMURAI‐NVAF registry and the collaboration with the joint initiative were approved by the ethics committee in the National Cerebral and Cardiovascular Center (M23‐18‐3 and M29‐077). CROMIS‐2 was approved by the National Research Ethics Committee, London (Queen Square). Patients with capacity gave informed written consent. When patients could not consent, we obtained written consent from a proxy as defined by relevant local legislation. 

### *Statistical Analysis Plan*

The statistical analysis was carried out using Stata (v14; StataCorp, College Station, Texas). The primary analysis was conducted in the entire cohort comparing patients with anticoagulation prior to admission (OAC_prior_) with those without anticoagulation on admission (OAC_naive_). OAC_prior_ was defined as a patient reporting to be on therapy with VKA or DOAC (apixaban, dabigatran, edoxaban, or rivaroxaban) at the time of onset of the index event. OAC_naive_ was defined as patients who were not on therapy with VKA or DOAC at the time of onset of the index event. OAC_naive_ included patients who were on antiplatelet agents or heparin.

The secondary analysis was conducted in the subgroup of patients with OAC_prior_. We compared patients in whom the type of anticoagulant was changed after the index event (OAC_changed_, the first oral anticoagulant the patient received after the index event) with those who continued the same type of anticoagulant after the event that they used already prior to having a stroke or TIA (OAC_unchanged_). OAC_changed_ was defined as one of the following: VKA prior to index event to DOAC after; DOAC prior to index event to VKA after; or changing type of DOAC (apixaban, dabigatran, edoxaban, or rivaroxaban). We excluded patients with severe renal impairment (CrCl < 30ml/min) from this subanalysis, as DOACs are contraindicated in these patients. We also excluded patients in whom the type of anticoagulation before or after the index event was not known.

We compared demographic and clinical baseline characteristics among groups using the Pearson χ^2^ test for categorical variables and the Mann–Whitney *U* test for continuous variables. We calculated the annualized rate of outcome events (total of observed events/patient‐years of follow‐up). For all analysis, we calculated time from starting first anticoagulation until first occurrence of an outcome event. In case of multiple events in the same patient, we only considered the time until first event. For the primary analysis, we constructed unadjusted cumulative incidence functions for outcomes and compared groups using the log‐rank test. We investigated the association between baseline characteristics and outcomes using univariate Cox proportional hazard regression models.

To further analyze the associations between groups and outcomes (AIS and ICH), we used multivariate competing risk models (competing risk: death) using the Fine–Gray model^27^ including the following prespecified variables: age; sex; history of stroke; diabetes mellitus; hypertension; NIHSS on admission; impaired renal function (CrCl < 50ml/min); diagnosis of AF (known before stroke vs diagnosed after stroke)^19^, ^20^, ^21^; and anticoagulation after index stroke (any anticoagulation vs no anticoagulation, only used in the primary analysis as all patients in the secondary analysis were using anticoagulants after the event). Additionally, shared frailty for study was introduced into all multivariate models to account for differences in local activity of care, resources, and ethnicity.

All analyses were conducted in the intention‐to‐treat population using the first prescribed oral anticoagulant after index stroke (ie, DOAC or VKA). We performed a post hoc sensitivity in the on‐treatment population including only patients with available information about changes in anticoagulant therapy during follow‐up. For sensitivity analyses, we used change in anticoagulation (ie, on treatment vs discontinuation) as a time‐varying covariate. We calculated hazard ratios (HRs) with 95% confidence intervals (CIs).

## Results

**Figure 1:**
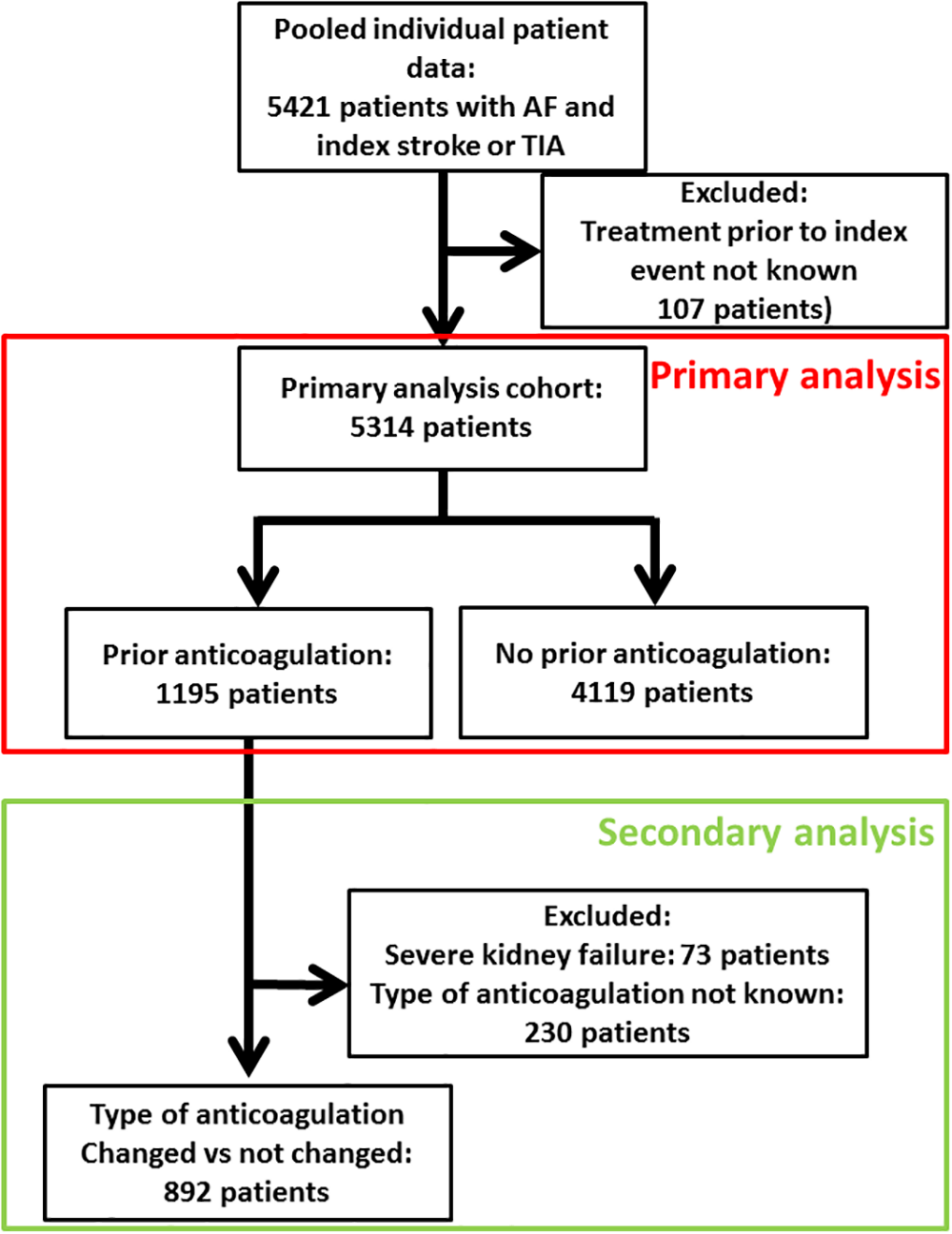
Study flow chart. AF = atrial fibrillation; TIA = transient ischemic attack. [Color figure can be viewed at www.annalsofneurology.org]

The final cohort for this analysis comprised 5,314 of 5,421 patients (98.0%) from the pooled data set of individual patient data (study flow chart in Fig 1). In this cohort, 2,559 patients (48%) were female, and 5,136 patients (96.7%) had an ischemic stroke as index event. The median age was 78 years (interquartile range [IQR] = 71–84 years), and the median NIHSS on admission was 6 (IQR = 2–12). Prior to the index event, 3,014 patients (56.7%) had received no antithrombotic treatment, 1,089 (20.5%) were on antiplatelets, 1,195 (22.5%) were on treatment with any oral anticoagulant (161 on DOAC, 865 on VKA, and in 169 the type of oral anticoagulation was not specified), and 16 (0.3%) were on other antithrombotic medications (ie, heparins). In patients on VKA prior to index stroke who had available data on INR (842 of 865 patients, 97.3%), the INR was <2.0 in 615 patients (73.0%), 2.0 to 3.0 in 185 patients (22.0%), and >3.0 in 42 patients (5.0%). After the index event, 4,929 patients (92.8%) were taking anticoagulants (DOAC in 2,716 patients; VKA in 2,213 patients), 359 patients (6.8%) were taking antiplatelet agents, and 26 patients (0.5%) had no antithrombotic therapy. For patients taking anticoagulants, the median delay between index event and (re)starting OAC was 5 days (IQR = 2–13). For patients using VKA after the event, information about time in therapeutic range was available in 924 of 2,213 patients (41.8%). Among these patients, 762 (82.5%) were recorded to have good (>60%) time in the therapeutic range. Among patients taking DOACs after the event, information on adherence was available from only 1 study (NOACISP^7^, ^26^) for 485 of 2,716 patients (17.6%). During follow‐up, 347 of these 485 patients (71.6%) were fully adherent. There was no difference in full adherence between patients that changed OAC after the index stroke and those who continued the same OAC (16 of 72 patients vs 28 of 108 patients; 77.8% vs 74.1% fully adherent, *p* > 0.05).

### *Primary Analysis: OAC_prior_* *vs OAC_naive_*

The baseline characteristics of patients with OAC_prior_ (n = 1,195) compared to OAC_naive_ (n = 4,119) are displayed in Table 1. Patients with OAC_prior_ were older, had a lower proportion of index ischemic stroke and a lower NIHSS on admission, and were less often treated with intravenous thrombolysis for the index event. They more often had a history of prior ischemic stroke before the index event, hypertension, hypercholesterinemia, diabetes mellitus, and modest or severe kidney failure. The CHA_2_DS_2_‐Vasc and HAS‐BLED scores did not differ between the OAC_prior_ and OAC_naive_ groups.

**Table 1:** Baseline Characteristics of Patients with and without Oral Anticoagulation Therapy prior to Index Event and Patients Who Did and Did Not Change the Type of Anticoagulation

	Primary Analysis			Secondary Analysis		
	OAC_prior_, n = 1,195	OAC_naive_, n = 4,119	*p*	OAC_changed_, n = 307	OAC_unchanged_, n = 585	*p*
Age^a^	79 (73–84)	77 (70–84)	<0.001	79 (74–84)	79 (72–83)	0.046
Female (%)	555/1,195 (46.4)	2,004/4,119 (48.7)	0.178	155/307 (50.6)	249/585 (42.6)	0.023
Prior treatment with VKA (%)	865/1,195 (72.4)	0	N/A	291/306 (95.1)	519/584 (88.8)	0.002
Ischemic stroke as index event (%)	1,144/1,195 (95.7)	3,992/4,119 (96.9)	0.045	229/307 (74.6)	519/585 (88.7)	0.002
History of ischemic stroke (other than index event) (%)	458/1,192 (38.4)	788/4,111 (19.2)	<0.001	119/306 (38.9)	222/583 (38.1)	0.828
History of ICH (%)	17/780 (2.2)	34/2,769 (1.2)	0.060	5/275 (1.8)	9/414 (2.2)	1.000
Hypertension (%)	1,026/1,195 (85.9)	2,958/4,089 (72.3)	<0.001	259/306 (84.6)	499/584 (85.4)	0.766
Hypercholesterinemia (%)	438/1,026 (42.7)	1,262/3,387 (37.3)	0.002	145/306 (47.4)	240/584 (41.1)	0.075
Smoking (%)	188/1,150 (16.3)	694/4,021 (17.3)	0.505	33/286 (11.5)	80/568 (14.1)	0.336
Diabetes mellitus (%)	442/1,194 (37.0)	890/4,109 (21.7)	<0.001	109/305 (35.7)	226/584 (38.7)	0.423
Normal renal function, CrCl > 50ml/min (%)	638/894 (71.4)	2,638/3,321 (79.4)	<0.001	207/273 (75.8)	105/305 (74.4)	0.719
Modest kidney failure, CrCl = 30–50ml/min (%)	185/894 (20.7)	554/3,321 (16.7)		66/273 (24.2)	105/410 (25.6)	
Severe kidney failure, CrCl < 30ml/min (%)	71/894 (7.9)	129/3,321 (3.9)		0	0	
Intravenous thrombolysis (%)	156/1,193 (13.1)	929/4,095 (22.7)	<0.001	51/304 (16.8)	60/584 (10.3)	0.007
Intraarterial treatment (%)	48/1,057(4.5)	141/3,897 (3.6)	0.174	15/278 (5.4)	18/483 (3.7)	0.274
NIHSS on admission^a^	5 (2–11)	6 (2–12)	<0.001	4 (2–10)	5 (2–11	0.222
CHA_2_DS_2_‐Vasc^a^	5 (4–6)	5 (4–6)	0.103	6 (4–6)	5 (4–6)	0.014
HAS‐BLED^a^	3 (3–4)	3 (3–4)	0.626	3 (2–4)	3 (3–4)	0.097

a Median (interquartile range).

CHA_2_DS_2_‐Vasc = congestive heart failure, hypertension, age ≥ 75 years, diabetes mellitus, stroke/transient ischemic attack, vascular disease, age 65–74 years, sex category; CrCl = creatinine clearance; HAS‐BLED = hypertension, abnormal renal/liver function, stroke, bleeding history or predisposition, labile international normalized ratio, elderly, drugs/alcohol; ICH = intracerebral hemorrhage; N/A = not applicable; NIHSS = National Institutes of Health Stroke Scale; OAC_changed_ = type of anticoagulant changed after index event; OAC_naive_ = no anticoagulation on admission; OAC_prior_ = anticoagulation prior to admission; OAC_unchanged_ = type of anticoagulant not changed after index event.

The total follow‐up time in the primary analysis data set of 5,314 patients was 6,128 patient‐years. During follow‐up, 289 patients had AIS (4.7% per year, 95% CI = 4.2–5.3%), 90 patients had ICH (1.5% per year, 95% CI = 1.2–1.8%), and 624 patients died (10.2% per year, 95% CI = 9.4–11.0%).

**Figure 2:**
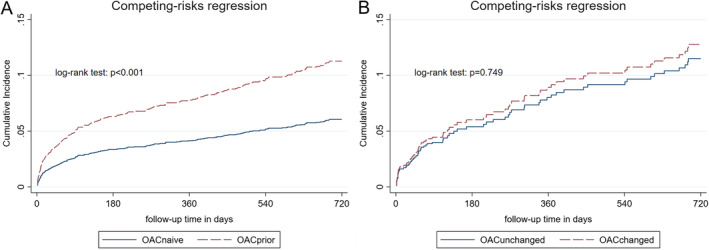
Cumulative incidence function curves for the main outcome of recurrent acute ischemic stroke. (A) Primary analysis of patients taking oral anticoagulation prior to the index event (OAC_prior_, dashed line) compared to those not taking anticoagulants prior to the index event (OAC_naive_, solid line). (B) Secondary analysis of patients that changed the type of anticoagulation (OAC_changed_, dashed line) compared to those who continued the same type of anticoagulation (OAC_unchanged_, solid line). [Color figure can be viewed at www.annalsofneurology.org]

Figure 2A shows cumulative incidence function curves for the primary outcome of recurrent AIS. OAC_prior_ had a higher rate of recurrent AIS (log‐rank test: *p* < 0.0001). There was little difference for ICH (log‐rank test: *p* = 0.425) and weak evidence for a higher risk of mortality (log‐rank test: *p* = 0.066). Table 2 displays annualized event rates for both groups and associations with outcomes: OAC_prior_ was associated with an increased risk of recurrent AIS both in univariate (HR = 2.0, 95% CI = 1.5–2.5, *p* < 0.001) and multivariate competing risk Fine–Gray analysis (HR = 1.6, 95% CI = 1.2–2.3, *p* = 0.005) but not with ICH or mortality.

**Table 2:** Observed and Annualized Rates of Outcome Events in Patients with OAC_prior_ and OAC_naive_ and Univariate and Multivariate Analysis

	OAC_naive_, n = 4,119		OAC_prior_, n = 1,195		Univariate		Multivariate^a^	
	Events, n^b^	Annualized Rate (95% CI)	Events, n^b^	Annualized Rate (95% CI)	Hazard Ratio (95% CI)	*p*	Hazard Ratio (95% CI)	*p*
AIS	196	3.9 (3.3–4.4)	93	8.9 (7.3–10.8)	2.0 (1.5–2.5)	<0.001	1.6 (1.2–2.3)	0.005
ICH	69	1.4 (1.0–1.7)	21	2.0 (1.3–3.1)	1.2 (0.7–2.0)	0.426	1.1 (0.5–2.3)	0.811
Mortality	501	9.9 (9.1–10.7)	123	11.8 (9.9–13.9)	1.2 (1.0–1.5)	0.069	1.1 (0.8–1.4)	0.667

a Multivariate competing risk Fine–Gray analysis was adjusted for the following prespecified variables: age, sex, history of ischemic stroke other than index event, hypertension, diabetes mellitus, modest or severe kidney failure (creatinine clearance < 50ml/min), diagnosis of atrial fibrillation (known before the ischemic stroke vs diagnosed after stroke), and treatment with any oral anticoagulant after index event. Study was introduced as a shared frailty term in this analysis.

b n = number of patients.

AIS = acute ischemic stroke; CI = confidence interval; ICH = intracerebral hemorrhage; OAC_naive_ no anticoagulation on admission; OAC_prior_ = anticoagulation prior to admission.

### *Secondary Analysis: OAC_changed_* *vs OAC_unchanged_*

Among the 1,195 patients with OAC_prior_, 892 patients were included in this secondary analysis (see study flow chart Fig 1), in whom type of anticoagulation prior to and after the index event was known and who did not have severe kidney failure (CrCl < 30ml/min). The baseline characteristics of patients with OAC_changed_ (n = 307) compared to OAC_unchanged_ (n = 585) are displayed in Table 1. Patients with OAC_changed_ were older, more often female, had more often an ischemic stroke as index event, and had a higher CHA_2_DS_2_‐VASc score. In the group of OAC_changed_, 229 patients (74.6%) changed from VKA to a DOAC, 26 patients (8.5%) changed from DOAC to VKA, and 52 patients (16.9%) changed the type of DOAC. In the group of OAC_unchanged_, 66 patients (11.3%) were on the same DOAC prior to and after the index event, and 519 patients (88.7%) were on VKA prior to and after the index event. In patients in the OAC_unchanged_ group that were on VKA prior to and after the index event, data on INR were available in 513 of 519 patients (98.8%), 358 patients (69.8%) had INR < 2.0, 132 patients (25.7%) had INR 2.0 to 3.0, and 23 patients (4.5%) had INR > 3.0 at index event.

The total follow‐up time included in the secondary analysis data set was 894 patient‐years. During follow‐up, 75 patients had AIS (8.4% per year, 95% CI = 6.7–10.4%), 17 patients had ICH (1.9% per year, 95% CI = 1.1–3.0%), and 85 patients died (9.5% per year, 95% CI = 7.7–11.6%) in this subanalysis.

Among patients on VKA (in both groups), data on INR at recurrent AIS during follow‐up were available in 26 of 42 patients (61.9%). In the OAC_unchanged_ group, the INR was <2.0 in 16 of 26 patients (61.5%) and ≥2.0 in 10 of 26 patients (38.5%) for patients on VKA. In the OAC_changed_ group, the INR at recurrent AIS was available for only 1 patient on VKA (INR <2.0).

Figure 2B displays cumulative incidence function curves for the primary outcome of recurrent AIS in the secondary analysis. OAC_changed_ was associated with a decreased risk of mortality in univariate (HR = 0.5, 95% CI = 0.3.–0.9, *p* = 0.012) but not multivariate analysis (HR = 0.7, 95% CI = 0.4–1.2, *p* = 0.177). We did not find any association of changing OAC with AIS or ICH (Table 3).

**Table 3:** Observed and Annualized Rates of Outcome Events in Patients with OAC_changed_ and OAC_unchanged_ and Univariate and Multivariate Analysis

	OAC_changed_, n = 307		OAC_unchanged_, n = 585		Univariate		Multivariate^a^	
	Events, n^b^	Annualized Rate (95% CI)	Events, n^b^	Annualized Rate (95% CI)	Hazard Ratio (95% CI)	*p*	Hazard Ratio (95% CI)	*p*
AIS	28	8.8 (5.9–12.4)	47	8.2 (6.1–10.7)	1.1 (0.7–1.7)	0.749	1.2 (0.7–2.1)	0.415
ICH	4	1.3 (0.3–3.2)	13	2.3 (1.2–3.8)	0.6 (0.2–1.8)	0.346	0.8 (0.2–3.2)	0.793
Mortality	19	5.9 (3.6–9.1)	66	11.5 (9.0–14.4)	0.5 (0.3–0.9)	0.012	0.7 (0.4–1.2)	0.177

a Multivariate competing risk Fine–Gray analysis was adjusted for the following prespecified variables: age, sex, history of ischemic stroke other than index event, hypertension, diabetes mellitus, diagnosis of atrial fibrillation (known before stroke vs diagnosed after stroke), and modest kidney failure (creatinine clearance = 30–50ml/min). Study was introduced as shared frailty term in this analysis.

b n = number of patients.

AIS = acute ischemic stroke; CI = confidence interval; ICH = intracerebral hemorrhage; OAC_changed_ = type of anticoagulant changed after index event; OAC_unchanged_ = type of anticoagulant not changed after index event.

### *Sensitivity Analysis: On‐Treatment Population*

We performed a post hoc analysis in the on‐treatment population (3,508 of 5,314 patients with available information on therapeutic changes during follow‐up; 66.0%). During follow‐up, 409 of 3,508 patients (11.7%) changed therapy: 122 patients changed the type of DOAC, 88 patients changed from DOAC to VKA, 157 patients changed from VKA to DOAC, and in 42 patients oral anticoagulation was discontinued. The results of the main analysis were confirmed in the on‐treatment population (HR for AIS = 1.8, 95% CI = 1.3–2.5, *p* < 0.001).

For the secondary analysis, information on changing anticoagulation during follow‐up was available in 689 of 892 patients (77.2%), of whom 129 patients (18.7%) changed therapy during follow‐up: 42 changed the type of DOAC, 25 changed from DOAC to VKA, 53 changed from VKA to DOAC, and 9 discontinued anticoagulation. The results of the main analysis were confirmed in the on‐treatment population (HR for AIS = 1.3, 95% CI = 0.7–2.1, *p* = 0.379).

## Discussion

This international collaborative study of 5,314 patients yielded the following main findings. First, 1,195 patients (22.5%) had an AIS or TIA despite treatment with oral anticoagulants. Second, these AF patients with prior anticoagulant use more often had cardiovascular risk factors compared to those who had not received anticoagulation at the time of the event, but had similar CHA_2_DS_2_‐Vasc and HAS‐BLED scores. Third, after adjusting for cardiovascular risk factors, patients who had a stroke despite treatment with oral anticoagulants were still at high risk of recurrent ischemic stroke. Fourth, changing the type of anticoagulant after the index event was not associated with a decreased risk of further ischemic strokes.

To the best of our knowledge, this is the first study addressing the question of whether patients failing anticoagulation therapy and having an ischemic stroke or TIA despite anticoagulant therapy are at subsequent high risk of further events. Both groups—those who had a stroke while on anticoagulant therapy and those without prior anticoagulation therapy—had a median CHA_2_DS_2_‐VASc score of 5, corresponding to an estimated risk of 7.2% per year of ischemic stroke.^28^ Nevertheless, the actual observed rate of recurrent ischemic strokes was twice as high in patients who had their index stroke despite taking anticoagulants (annualized rate = 8.9%, 95% CI = 7.3–10.8%) compared to those who were not on anticoagulation therapy at the time of index stroke (annualized rate = 3.9%, 95% CI = 3.3–4.4%). This raises the key questions of why patients with OAC_prior_ are at increased risk and what the optimal prevention strategy might be to reduce this risk.

One potential explanation is nonadherence to prescribed anticoagulation therapy prior to the index event. A majority (73%) of patients on VKA prior to the index event had subtherapeutic INR, indicating poor adherence. Among those patients who continued VKA after the event, 61% of further recurrent ischemic strokes occurred at subtherapeutic INR values. In these patients, poor adherence might thus have played a role, even though they all had already suffered from a significant outcome event—ischemic stroke. However, patients who start on DOACs for secondary prevention generally achieve high rates of adherence^26^ making this unlikely to be a full explanation. Furthermore, patients who were OAC naive prior to a stroke were reported to have lower rates of adherence^26^ than those who had used anticoagulants in the past, which would bias in the opposite direction from our findings. In addition, a recent analysis in a study from Japan found that in patients taking VKA, an INR of ≥2.0 at stroke onset was associated with a higher risk of recurrent ischemic stroke.^29^

Genetic variability could be a cause of susceptibility to recurrent stroke in patients with AF: 2 genes (CYP2C9 and VKORC1) may play a role in individual patient response to and efficacy of VKA,^30^, ^31^, ^32^, ^33^ but no such variability of response is known for DOACs. However, most patients who changed anticoagulation after the event were switched from VKA to a DOAC (76%), so a genetic variability in 1 of the aforementioned genes is not likely to explain the high continued ischemic stroke risk we observed.

Alternatively, the prior use of anticoagulants might be for pulmonary embolism or deep vein thrombosis. These conditions may have been present independent of AF and could put patients at a higher stroke risk than patients with pure AF, for example due to a prothrombotic state, paradoxical embolism of increased inflammatory state. Further monitoring (eg, anti‐Xa levels) could be useful in this population.

Our data show that despite similar median CHA_2_DS_2_‐VASc scores, several cerebrovascular risk factors (hypertension, diabetes, hypercholesterinemia, kidney failure) were more frequent in patients with prior anticoagulation. Furthermore, 38% of patients on prior anticoagulation had already experienced an ischemic stroke before the index event in this study. This may point toward competing stroke risk factors and stroke etiologies—for example, large artery atherosclerosis or small vessel disease—that might be less responsive to anticoagulation therapy.^2^ Further research needs to focus on mechanisms, competing risk factors, and etiology of (recurrent) stroke in patients taking anticoagulants. This could include taking into account competing causes as in the ASCO^34^ or ASCOD^35^ (A = atherosclerosis, S = small‐vessel disease, C = cardiac pathology, O = other causes, D = dissection) causality score systems.

This hypothesis is further supported by our secondary analysis, which found that simply changing the type of anticoagulation was not associated with a reduced risk of ischemic stroke. We do not have data on the presence of concomitant stroke etiologies, some of which, such as small vessel disease, might not respond well to anticoagulants and could contribute to our results. However, other mechanisms including off‐label low‐dose use of DOAC, which has been described as a potential cause of ischemic stroke despite DOAC therapy in prior studies,^36^ could have played a role.

For those patients with recurrent cardioembolic strokes, more research is needed to determine whether the dynamics of anticoagulant activity^37^ or the different types of anticoagulants and their pharmacokinetics (eg, a long, sustained effect for VKA, a 2–4 hour high peak effect every 24 hours for once‐daily edoxaban and rivaroxaban, or every 12 hours for twice‐daily apixaban and dabigatran) affect recurrence in these patients. Moreover, DOAC switching might deserve further prospective investigation in larger study populations given that network meta‐analyses of available randomized controlled trials have provided indirect evidence of differential safety and efficacy profiles among available DOACs.^38^, ^39^

Regardless of the underlying explanation, our findings have clearly identified patients with AF who have ischemic stroke despite oral anticoagulation as a group at high risk of subsequent recurrence who require better prevention strategies. Recent data suggest that combination DOAC and antiplatelet therapy might be effective for stroke prevention.^40^, ^41^ Combination strategies, for example adding antiplatelet therapy or left atrial appendage occlusion to oral anticoagulation might merit further investigation in controlled trials in patients with AF who have “failed” oral anticoagulation by sustaining an ischemic stroke.

Our study has the following strengths: (1) we included data from 7 international studies involving patients from Europe and Asia (and 1 center in North America), which makes our results broadly generalizable worldwide; (2) we report on a large, stroke‐unit based data set of patients with a recent ischemic stroke and AF with more than 5,000 patients and more than 6,000 patient‐years of follow‐up; (3) all participating studies prospectively recruited stroke patients (in the majority of cases consecutively), minimizing selection bias; and (4) we included patients at high risk for both ischemic stroke and intracranial bleeding, with a median CHA_2_DS_2_‐VASc score of 5 and a HAS‐BLED score of 3. Ninety‐three percent of our patients received any oral anticoagulation after the index event, which suggests that the majority of patients received best medical therapy to prevent further events.

Our study has some limitations: (1) we report on an observational study rather than a randomized trial, and allocation to the type of oral anticoagulant is likely to be affected by biases (including physician factors) that cannot be fully adjusted for; (2) OAC_prior_ was determined by history only, and the time of last OAC intake was not available; (3) we did not record the reason for OAC_prior_ or the etiology of index or follow‐up strokes, and so can only speculate on mechanisms of recurrent stroke; (4) we could not determine whether patients pretreated with DOACs received the appropriate dose based on their age, body weight, and creatinine or estimated glomerular filtration rate levels; (5) although the largest cohort of patients reported with stroke while on treatment with anticoagulation, the sample size was insufficient to perform in‐depth analysis of different strategies to prevent further events, and in particular was not powered to investigate whether any specific type of anticoagulant is associated with a decreased risk for further events; (6) we did not have data on concomitant atherosclerosis and small vessel disease and other secondary prevention strategies (statins, hypertensive agents) or other factors related to confounding by indication (eg, hemorrhagic transformation, prior dementia, institutionalization, lack of social support, etc, which could influence physicians’ decisions to prescribe OAC); and (7) although one might expect patients switched to a DOAC to have a lower rate of ICH, this was not the case in this analysis, as the subgroup analysis was not powered for this endpoint. However, in our prior analysis,^4^ patients taking DOACs after a recent ischemic stroke had a significantly lower risk of ICH compared to those taking VKA.

To summarize, we found that patients having a stroke despite being on therapy with an oral anticoagulant are at high risk of recurrent ischemic strokes. Further research is needed to investigate mechanisms of recurrent stroke and improve secondary prevention in these patients.

## Authors Contributions

D.S., G.M.D.M., and D.J.W. conceived the study and drafted the manuscript. D.S. performed the statistics. All authors contributed to the design of the studies, collection and interpretation of the data, and editing the manuscript.

## Potential Conflicts of Interest

The following companies manufacture drugs involved in this study: Bayer (BY), Boehringer Ingelheim (BI), Pfizer/Bristol Meyer Squibb (PB), and Daiichi Sankyo (DSA). D.S.: scientific advisory boards, BY and PB. G.M.D.M.: travel honoraria, BY; speaker honoraria, PB. M.P.: honoraria for speaker bureau, BY, BI, PB, and DSA. K.Ma.: advisory boards, BI, BY, and DSA. S.T.E.: travel or speaker honoraria, BY, BI; scientific advisory boards, BY, BI, and PB; educational grant, PB. D.J.W.: speaking honoraria, BY. G.T.: speaking and advisory board honoraria, BI, DSA, and BY. All other authors have nothing to report.

## Supporting information


**Appendix** **S1:** Supporting information
