# Supplementary material for: Ischemic Stroke despite Oral Anticoagulant Therapy in Patients with Atrial Fibrillation
Source: Ann Neurol. 2020 Feb 27;87(5):677–87. doi: 10.1002/ana.25700 (PMC7383617; doi:10.1002/ana.25700)
Supplement: Supplementary file 1 — Appendix S1: Supporting information [file ANA-87-677-s001.docx]

**Group authors:**

| Name | Institution |
| --- | --- |
| ***SAMURAI-NVAF*** | |
| Kenichi Todo MD | Osaka University Graduate School of Medicine |
| Kazumi Kimura MD | Kawasaki Medical School (Nippon Medical School) |
| Kensaku Shibazaki MD | Kawasaki Medical School |
| Yoshiki Yagita MD | Kawasaki Medical School |
| Eisuke Furui MD | Kohnan Hospital |
| Ryo Itabashi MD | Kohnan Hospital |
| Tadashi Terasaki MD | Japanese Red Cross Kumamoto Hospital |
| Yoshiaki Shiokawa, MD | Kyorin University School of Medicine |
| Teruyuki Hirano MD | Kyorin University School of Medicine |
| Rieko Suzuki MD | Kyorin University School of Medicine |
| Kenji Kamiyama MD | Nakamura Memorial Hospital |
| Jyoji Nakagawara MD | Nakamura Memorial Hospital |
| Shunya Takizawa MD | Tokai University School of Medicine |
| Kazunari Homma MD | Tokai University School of Medicine |
| Satoshi Okuda MD | NHO Nagoya Medical Center |
| Yasushi Okada MD | NHO Kyushu Medical Center |
| Koichiro Maeda MD | NHO Kyushu Medical Center |
| Tomoaki Kameda MD | Jichi Medical University School of Medicine |
| Kazuomi Kario MD | Jichi Medical University School of Medicine |
| Yoshinari Nagakane MD | Kyoto Second Red Cross Hospital |
| Yasuhiro Hasegawa MD | St Marianna University School of Medicine |
| Hisanao Akiyama MD | St Marianna University School of Medicine |
| Satoshi Shibuya MD | South Miyagi Medical Center |
| Hiroshi Mochizuki MD | South Miyagi Medical Center |
| Yasuhiro Ito MD | TOYOTA Memorial Hospital |
| Takahiro Nakashima MD | NHO Kagoshima Medical Center |
| Hideki Matsuoka MD | NHO Kagoshima Medical Center |
| Kazuhiro Takamatsu MD | Brain Attack Center Ota Memorial Hospital |
| Kazutoshi Nishiyama MD | Kitasato University School of Medicine |
| Kanta Tanaka MD | National Cerebral and Cardiovascular Center |
| Kaoru Endo MD | National Cerebral and Cardiovascular Center |
| Tetsuya Miyagi MD | National Cerebral and Cardiovascular Center |
| Masato Osaki MD | National Cerebral and Cardiovascular Center |
| Junpei Kobayashi MD | National Cerebral and Cardiovascular Center |
| Takuya Okata MD | National Cerebral and Cardiovascular Center |
| Eijiro Tanaka MD | National Cerebral and Cardiovascular Center |
| Yuki Sakamoto MD | National Cerebral and Cardiovascular Center |
| Keisuke Tokunaga MD | National Cerebral and Cardiovascular Center |
| Hotake Takizawa MD | National Cerebral and Cardiovascular Center |
| Junji Takasugi MD | National Cerebral and Cardiovascular Center |
| Soichiro Matsubara MD | National Cerebral and Cardiovascular Center |
| Kyoko Higashida MD | National Cerebral and Cardiovascular Center |
| Takayuki Matsuki MD | National Cerebral and Cardiovascular Center |
| Naoto Kinoshita MD | National Cerebral and Cardiovascular Center |
| Masayuki Shiozawa MD | National Cerebral and Cardiovascular Center |
| Toshihiro Ide MD | National Cerebral and Cardiovascular Center |
| Takeshi Yoshimoto MD | National Cerebral and Cardiovascular Center |
| Daisuke Ando MD | National Cerebral and Cardiovascular Center |
| Kyohei Fujita MD | National Cerebral and Cardiovascular Center |
| Masaya Kumamoto MD | National Cerebral and Cardiovascular Center |
| Teppei Kamimura MD | National Cerebral and Cardiovascular Center |
| Muneaki Kikuno MD | National Cerebral and Cardiovascular Center |
| Tadataka Mizoguchi MD | National Cerebral and Cardiovascular Center |
| Takeo Sato MD | National Cerebral and Cardiovascular Center |
| ***RAF and RAF-DOAC*** | |
| Karen L Furie MD | Division of Stroke and Cerebrovascular Diseases, Department of Neurology, The Warren Alpert Medical School of Brown University, Providence, RI, USA |
| Prasanna Tadi MD | Division of Stroke and Cerebrovascular Diseases, Department of Neurology, The Warren Alpert Medical School of Brown University, Providence, RI, USA |
| Cecilia Becattini MD | Stroke Unit and Division of Cardiovascular Medicine, University of Perugia, Italy |
| Nicola Falocci PhD | Stroke Unit and Division of Cardiovascular Medicine, University of Perugia, Italy |
| Marialuisa Zedde MD | Neurology Unit, Stroke Unit, Arcispedale Santa Maria Nuova, Azienda Unità Sanitaria Locale – IRCCS, Reggio Emilia, Italy |
| Azmil H Abdul-Rahim MD | Medical School and Institute of Cardiovascular and Medical Sciences, University of Glasgow, Glasgow, United Kingdom. |
| Kennedy R Lees MD | Medical School and Institute of Cardiovascular and Medical Sciences, University of Glasgow, Glasgow, United Kingdom. |
| Cataldo D’Amore MD | Stroke Unit and Division of Cardiovascular Medicine, University of Perugia, Italy |
| Maria G Mosconi MD | Stroke Unit and Division of Cardiovascular Medicine, University of Perugia, Italy |
| Ludovica A Cimini MD | Stroke Unit and Division of Cardiovascular Medicine, University of Perugia, Italy |
| Paolo Bovi MD | SSO Stroke Unit, UO Neurologia, DAI di Neuroscienze, AOUI Verona, Italy |
| Monica Carletti MD | SSO Stroke Unit, UO Neurologia, DAI di Neuroscienze, AOUI Verona, Italy |
| Alberto Rigatelli MD | SSO Stroke Unit, UO Neurologia, DAI di Neuroscienze, AOUI Verona, Italy |
| Manuel Cappellari MD | SSO Stroke Unit, UO Neurologia, DAI di Neuroscienze, AOUI Verona, Italy |
| Jukka Putaala MD | Department of Neurology, Helsinki University Central Hospital, Helsinki, Finland |
| Liisa Tomppo MD | Department of Neurology, Helsinki University Central Hospital, Helsinki, Finland |
| Turgut Tatlisumak MD | Department of Clinical Neuroscience, Institute of Neuroscience and Physiology, Sahlgrenska Academy at University of Gothenburg and Department of Neurology, Sahlgrenska University Hospital, Gothenburg, Sweden |
| Fabio Bandini MD | Department of Neurology, Ospedale San Paolo, Savona, Italy |
| Simona Marcheselli MD | Neurologia d'urgenza e Stroke Unit, Istituto Clinico Humanitas, Rozzano, Milano, Italy |
| Alessandro Pezzini MD | Department of Clinical and Experimental Sciences, Neurology Unit, University of Brescia, Italy |
| Loris Poli MD | Department of Clinical and Experimental Sciences, Neurology Unit, University of Brescia, Italy |
| Alessandro Padovani MD | Department of Clinical and Experimental Sciences, Neurology Unit, University of Brescia, Italy |
| Luca Masotti MD | Internal Medicine, Santa Maria Nuova Hospital, Firenze, Italy |
| Vieri Vannucchi MD | Internal Medicine, Santa Maria Nuova Hospital, Firenze, Italy |
| Sung-Il Sohn MD | Department of Neurology, Keimyung University School of Medicine, Daegu, South Korea |
| Gianni Lorenzini MD | SC Medicina e Chirurgia d’Accettazione e d’Urgenza, Ospedale Lotti Pontedera, Azienda USL Toscana Nordovest |
| Rossana Tassi MD | Stroke Unit, AOU Senese, Siena, Italy |
| Francesca Guideri MD | Stroke Unit, AOU Senese, Siena, Italy |
| Maurizio Acampa MD | Stroke Unit, AOU Senese, Siena, Italy |
| Giuseppe Martini MD | Stroke Unit, AOU Senese, Siena, Italy |
| George Ntaios MD | Department of Medicine, University of Thessaly, Larissa, Greece |
| Efstathia Karagkiozi MD | Department of Medicine, University of Thessaly, Larissa, Greece |
| George Athanasakis MD | Department of Medicine, University of Thessaly, Larissa, Greece |
| Kostantinos Makaritsis MD | Department of Medicine, University of Thessaly, Larissa, Greece |
| Kostantinos Vadikolias MD | Department of Neurology, Democritus University of Thrace, University Hospital of Alexandroupolis, Greece |
| Chrysoula Liantinioti MD | Second Department of Neurology, “Attikon” University Hospital, National & KapodistrianUniversity of Athens, School of Medicine, Athens, Greece |
| Maria Chondrogianni MD | Second Department of Neurology, “Attikon” University Hospital, National & KapodistrianUniversity of Athens, School of Medicine, Athens, Greece |
| Nicola Mumoli MD | Department of Internal Medicine, Ospedale Civile di Livorno, Italy |
| Domenico Consoli MD | Stroke Unit, Jazzolino Hospital, Vibo Valentia, Italy |
| Franco Galati MD | Stroke Unit, Jazzolino Hospital, Vibo Valentia, Italy |
| Simona Sacco MD | Department of Neurology, University of L’Aquila, Avezzano Hospital, Italy |
| Antonio Carolei MD | Department of Neurology, University of L’Aquila, Avezzano Hospital, Italy |
| Cindy Tiseo MD | Department of Neurology, University of L’Aquila, Avezzano Hospital, Italy |
| Francesco Corea MD, PhD | UO Gravi Cerebrolesioni, San Giovanni Battista Hospital, Foligno |
| Walter Ageno MD | Department of Internal Medicine, Insubria University, Varese, Italy |
| Marta Bellesini MD | Department of Internal Medicine, Insubria University, Varese, Italy |
| Giorgio Silvestrelli MD, PhD | S.C. di Neurologia e S.S. di Stroke Unit, ASST di Mantova, Mantova, Italy |
| Alfonso Ciccone MD | S.C. di Neurologia e S.S. di Stroke Unit, ASST di Mantova, Mantova, Italy |
| Umberto Scoditti MD | Stroke Unit, Neuroscience Department, University of Parma, Italy |
| Licia Denti MD | Stroke Unit - Dipartimento Geriatrico Riabilitativo – University of Parma, Italy |
| Michelangelo Mancuso MD | Clinica Neurologica – Azienda Ospedaliero-Universitaria, Pisa, Italy |
| Miriam Maccarrone MD | Clinica Neurologica – Azienda Ospedaliero-Universitaria, Pisa, Italy |
| Giovanni Orlandi MD | Neurologia, Ospedale Apuano, Massa Carrara, Italy |
| Nicola Giannini MD | Neurologia, Ospedale Apuano, Massa Carrara, Italy |
| Gino Gialdini MD | Clinica Neurologica – Azienda Ospedaliero-Universitaria, Pisa, Italy |
| Tiziana Tassinari MD | Stroke Unit-Department of Neurology, Santa Corona Hospital, Pietra Ligure (Savona), Italy |
| Maria Luisa De Lodovici MD | Stroke Unit, Neurology, Insubria University, Varese, Italy |
| Giorgio Bono MD | Stroke Unit, Neurology, Insubria University, Varese, Italy |
| Christina Rueckert MD | Abteilung für Neurologie, Oberschwabenklinik gGmbH, Ravensburg, Germany |
| Antonio Baldi MD | Stroke Unit, Ospedale di Portogruaro, Portogruaro (Venice), Italy |
| Danilo Toni MD, PhD | Department of Neurology and Psychiatry, Sapienza University of Rome, Italy |
| Federica Letteri MD | Department of Neurology and Psychiatry, Sapienza University of Rome, Italy |
| Martina Giuntini MD | Neurologia, Ospedale Apuano, Massa Carrara, Italy |
| Enrico M Lotti MD | U.O. Neurologia Presidio Ospedaliero di Ravenna Azienda USL della Romagna, Italy |
| Yuriy Flomin MD | Stroke and Neurorehabilitation Unit MC ‘Universal Clinic ‘Oberig’ Kyiv, Ukraine |
| Alessio Pieroni MD | Department of Neurology and Psychiatry, Sapienza University of Rome, Italy |
| Odysseas Kargiotis MD | Stroke Unit, Metropolitan Hospital, Piraeus, |
| Theodore Karapanayiotides MD, PhD | 2^nd^ Department of Neurology, AHEPA University Hospital, Thessaloniki, Greece |
| Serena Monaco MD | Stroke Unit, Ospedale Civico, Palermo, Italy |
| Laszló Csiba MD | Stroke Unit, University of Debrecen, Hungary |
| Lilla Szabó MD | Stroke Unit, University of Debrecen, Hungary |
| Alberto Chiti MD | Divisione di Neurologia, Ospedale Galliera, Genoa, Italy |
| Elisa Giorli MD | Divisione di Neurologia, Ospedale Galliera, Genoa, Italy |
| Massimo Del Sette MD | Divisione di Neurologia, Ospedale Galliera, Genoa, Italy |
| Davide Imberti MD | Department of Internal Medicine, Ospedale Civile di Piacenza, Italy |
| Dorjan Zabzuni MD | Department of Internal Medicine, Ospedale Civile di Piacenza, Italy |
| Boris Doronin MD | Municipal Budgetary Healthcare Institution of Novosibirsk. City Clinical Hospital # 1. Novosibirsk (Russia) at the Novosibirsk State Medical University (Russia) |
| Vera Volodina MD | Municipal Budgetary Healthcare Institution of Novosibirsk. City Clinical Hospital # 1. Novosibirsk (Russia) at the Novosibirsk State Medical University (Russia) |
| Patrik Michel, PD-MER | Centre Cérébrovasculaire, Service de Neurologie, Département des Neurosciences Cliniques Centre Hopitalier Universitaire Vaudois, Lausanne (Switzerland) |
| Peter Vanacker MD | Department of Neurology, Born Bunge Institute, Antwerp University Hospital, Antwerp, Belgium |
| Kristian Barlinn MD | Department of Neurology, Dresden University Stroke Center, Dresden, Germany |
| Lars P Pallesen MD | Department of Neurology, Dresden University Stroke Center, Dresden, Germany |
| Ulf Bodechtel MD | Department of Neurology, Dresden University Stroke Center, Dresden, Germany |
| Leonardo Ulivi MD | Clinica Neurologica – Azienda Ospedaliero-Universitaria, Pisa, Italy |
| Dirk Deleu, MD, PhD | Neurology, Hamad Medical Corporation, Doha, Qatar |
| Gayane Melikyan MD | Neurology, Hamad Medical Corporation, Doha, Qatar |
| Jessica Bourlinn, MD | Neurology, Hamad Medical Corporation, Doha, Qatar |
| Naveed AKhar, MD, | Neurology, Hamad Medical Corporation, Doha, Qatar |
| Falsal Ibrahin, MD | Neurology, Hamad Medical Corporation, Doha, Qatar |
| Gourbali Vanessa, MD | Department of Neurology, Evangelismos Hospital, Athens |
| Shadi Yaghi, MD | Division of Stroke and Cerebrovascular Diseases, Department of Neurology, The Warren Alpert Medical School of Brown University, Providence, RI, USA |
| Hawone Baronello, MD | Stroke Unit, Ospedale Civico, Palermo, Italy |
| ***NOACISP/Basel*** | |
| Lisa Hert | University Hospital Basel, Switzerland |
| Nils Peters | University Hospital Basel, Switzerland |
| Marina Maurer | University Hospital Basel, Switzerland |
| Martina Wiegert | University Hospital Basel, Switzerland |
